# To replace or not to replace the aortic valve during Type A Aortic Dissection surgery: early and mid-term results

**DOI:** 10.3389/fcvm.2025.1543017

**Published:** 2025-06-19

**Authors:** Muhammet Selim Yaşar, Emre Külahcıoğlu, Şeref Alp Küçüker

**Affiliations:** ^1^ Department of Cardiovascular Surgery, Mardin Education and Research Hospital, Mardin, Türkiye; ^2^ Department of Cardovascular Surgery, Kilis Prof. Dr. Alaeddin Yavaşca State Hospital, Kilis, Türkiye; ^3^ Department of Cardiovascular Surgery, Ankara Bilkent City Hospital, Ankara, Türkiye

**Keywords:** aortic valve replacement, supracoronary graft interposition, modified bentall procedure, AV resuspension, TYPE A AORTIC DISSECTION (TAAD)

## Abstract

**Aim:**

The decision to replace or not to replace the aortic valve in the surgical treatment of Type A Aortic Dissection can be complicated in hesitant cases. It is controversial which procedure should be used in such cases and may significantly alter intraoperative/postoperative patient care and disease prognosis in an operation with a high mortality and morbidity rate, such as Type Aortic Dissection Surgery. In this study, we aim to compare the early and mid-term results of these two different methods.

**Methods:**

Between February 2019 and September 2022, 112 consecutive patients examined who underwent operation for TYPE A AORTIC DISSECTION in our clinic, retrospectively. Patients were than divided into two groups: those who the valve replaced group (Modified Bentall Procedure, SGI + AVR), (*n* = 26, 23.2%), and those who had the not valve replaced group (Isolated SGI, David II procedure, AV Resuspension), (*n* = 86, 76.8%).

**Results:**

It was observed that the X-Clamp and CPB times were longer and the need for postoperative mechanical support was higher in the valve replaced group (*p* < 0.05). Although it was not statistically significant, the false lumen patency rate was higher and the survival time was lower in the valve replaced group. In the postoperative controls, moderate-to-severe aortic regurgitation was not seen in any of the patients who had preoperative moderate-to-severe aortic regurgitation in the not valve replaced group and there was no sinus valsalva aneurysm in any patient.

**Conclusion:**

When the intraoperative and postoperative results in our study were evaluated, it was concluded that the not valve replaced was superior to the valve replaced procedures for TYPE A AORTIC DISSECTION patients.

## Introduction

Aortic dissection is strongly associated with increased mortality if left untreated. According to the International Registry of Acute Aortic Dissection (IRAD), mortality rates increase significantly when acute dissections involving the proximal aorta are not treated surgically. In-hospital mortality for patients treated medically is 58% compared to 26% for those undergoing surgical intervention. Therefore, prompt surgical intervention is recommended for such cases (1).

The strategic course of the surgical procedure can vary markedly depending on a multitude of factors including the location of the dissecting flap, the age of the patient, the presence or absence of comorbidity, connective tissue disease, degree of aortic regurgitation, and the experience of the surgical team and hospital center (2). In particular, whether or not to perform valve replacement in patients with severe aortic regurgitation but physically intact leaflet tissue is a matter of debate. Studies demonstrate that, for such cases, aortic valve resuspension and valve-sparing root surgery can be employed (3–7). Even for “above-the-coronaries” procedures — which directly affect functional aortic anatomy like isolated supracoronary graft interposition — may improve severe aortic regurgitation despite not intervening in the aortic root (4–8). However, some surgeons are hesitant to follow a valve preservation strategy due to the risk of residual aortic insufficiency and future aneurysm development requiring reoperation in the sinus region (7–9). For patients undergoing mechanical valve replacement, the postoperative anticoagulation strategy changes. This could contribute to continuous false lumen patency post-operation and, consequently, the development of aneurysmal dilatation in the non-operated parts of the aorta.

Although the extent of repair in the ascending aorta has a direct impact on the aortic valve, the more important decision, which is related to all of the above-mentioned question marks, is whether or not to perform valve replacement. This study investigates early and mid-term outcomes of the valve replaced vs. the not valve replaced patients undergoing TYPE A AORTIC DISSECTION repair, and delineates the differences in outcomes.

## Methods

Between February 2019 and September 2022, 112 consecutive patients underwent operation for TYPE A AORTIC DISSECTION in our clinic. Preoperative, intraoperative, and postoperative variables were examined retrospectively from patient files. Patients were than divided into two groups: those the valve replaced group (*n* = 26, 23.2%), and those the not valve replaced group (*n* = 86, 76.8%).

In this study, the primary outcome variables were determined as early or mid-term death and need for reoperation (e.g., severe aortic insufficiency, development of dissecting aneurysm, etc.). Secondary outcome variables were determined as mid-term false lumen patency/thrombosis and proximal or distal aortic diameter increase.

The institutional ethical board granted approval for this study (Ankara Bilkent City Hospital E.K.-E1-22-2510, No: 2510). Written consent was obtained from all of the patients.

## Statistical method

Data analysis was conducted using IBM SPSS 25.0 (Armonk, NY: IBM Corp.) and MedCalc 15.8 (MedCalc Software bvba, Ostend, Belgium). Descriptive statistical methods (frequency, percentage, mean, standard deviation, median, min-max), as well as the chi-square test for comparing qualitative data, were used when evaluating the study data. Compatibility of data with normal distribution was evaluated with the Kolmogorov–Smirnov test, skewness-kurtosis, and graphical methods (histogram, Q-Q Plot, Stem and Leaf, Boxplot). In the study, different tests were used to compare the quantitative data: for normally distributed data, between groups using Independent Samples *t*-test, within groups using Paired Samples *t*-test or Repeated Measures Anova; and for non-normally distributed data, between groups using the Mann–Whitney *U* test, and within groups using the Wilcoxon test or Friedman test. Survival and life analyses were performed using the Kaplan–Meier, Log Rank, Breslow, and Tarone-Ware tests. The statistical significance level accepted as *p* = 0.05.

Power analysis was conducted using the G*Power 3.1.9.7 statistics package (Franz Faul, University of Kiel, Germany). With n1 = 26 (59.6 ± 10.1), n2 = 86 (48.0 ± 6.9), Effect Size (d) = 1.26, *α* = 0.05; power was found to be 99%.

## Results

For the entire cohort, 83 patients (74.1%) were male and 29 patients (25.9%) were female. Average age was 54.8 ± 12.2 years [median 56.5 years (range 25.0–79.0 years)]. For preoperative characteristics, 67 patients (59.8%) had a history of hypertension, 28 patients (25%) were smoker, 3 patients (2.6%) were diagnosed with Marfan's syndrome, 4 patients (3.6%) were under observation due to ascending aortic aneurysm, 2 patients (1.7%) underwent surgery with cardiopulmonary resuscitation. Most patients presented with symptoms of chest and/or back pain (*n* = 90, 80.3%). 11 patients (9.8%) presented with syncopy. One patient (0.9%) was operated on due to iatrogenic aortic dissection after coronary angiography. Lower extremity pulse loss was observed in 20 patients (17.8%). Various degrees of altered consciousness, ranging from confusion to coma, were observed in 6 patients (5.4%) (Table 1).

**Table 1:** Demographics of patients.

	*n*	%
	Avg. ± SD	Median (Min-Max)
Gender*		
Female	29	25.9
Male	83	74.1
Age (year)**	54.8 ± 12.2	56.5 (25.0–79.0)
Comorbidities*		
Hypertension	67	59.8
Current tobacco use	28	25.0
Diabetes	5	4.5
Marfan syndrome	3	2.6
Known ascending aortic aneurysm	4	3.6
With Cardiopulmonary resuscitation	2	1.7

* *n*/%.

** Average ± Standart Deviation/Median (Min-Max).

Total cohort were divided into two groups. The first group (n:26) comprised of 25 patients who underwent the Modified Bentall procedure and one patient who received Supracoronary Graft Interposition (SGI) with aortic valve replacement (AVR). All patients who received the Modified Bentall procedure had moderate or severe preoperative aortic regurgitation on echocardiography, and/or the dissection flap included both the sinus of Valsalva and at least one of the aortic commissures.

The second group (n:86) consisted of six patients who underwent SGI with aortic valve resuspension with commissural pledgeted sutures, 2 patients who underwent Valve-Sparing Root Replacement (David II Procedure), and 78 patients who had isolated supracoronary graft interposition (Table 2).

**Table 2:** Groups of operative procedures.

	*n*	%
The not valve replaced group	86	76.8
Isolated SKI	78	69.6
SKI + AV Resuspension	6	5.4
David II procedure	2	1.8
The valve replaced group	26	23.2
Modified Bentall Procedure	25	22.3
SKG + AVR	1	0.9

Preoperatively on echocardiographic examination, moderate-to-severe aortic regurgitation was found in 40.2% (*n* = 45) of the total cohort. Of these 45 patients, valve replacement was carried out in 42.2% (*n* = 19) during surgery, while valve replacement was not required in 57.8% (*n* = 26). For the entire cohort most patients (*n* = 86, 76.8%) did not require valve replacement. No patient was found with moderate to severe aortic regurgitation in postoperative follow-ups, even if they had preoperative moderate-severe aortic regurgitation. It was found that patients with bicuspid aortic valve (*n* = 9, 8%) were evenly distributed between the groups (Table 3).

**Table 3:** Echocardiographic examination.

		*n*	%
		Avg. ± SD	Median (Min-Max)
Ejektion Fraction**	Preoperative	57.1 ± 6.0	60.0 (27.0–65.0)
	Postoperative	54.1 ± 7.3	55.0 (30.0–65.0)
Aortic Regurgitation (Preoperative)*	Mild	57	50.9
	Moderate	27	24.1
	Severe	18	16.1
Aortic Regurgitation (Postoperative)*	Mild	71	63.4
Leaflet characteristic*	Bicuspid	9	8.0

* *n*/%.

** Average ± Standart Deviation/Median (Min-Max).

**Figure 1:**
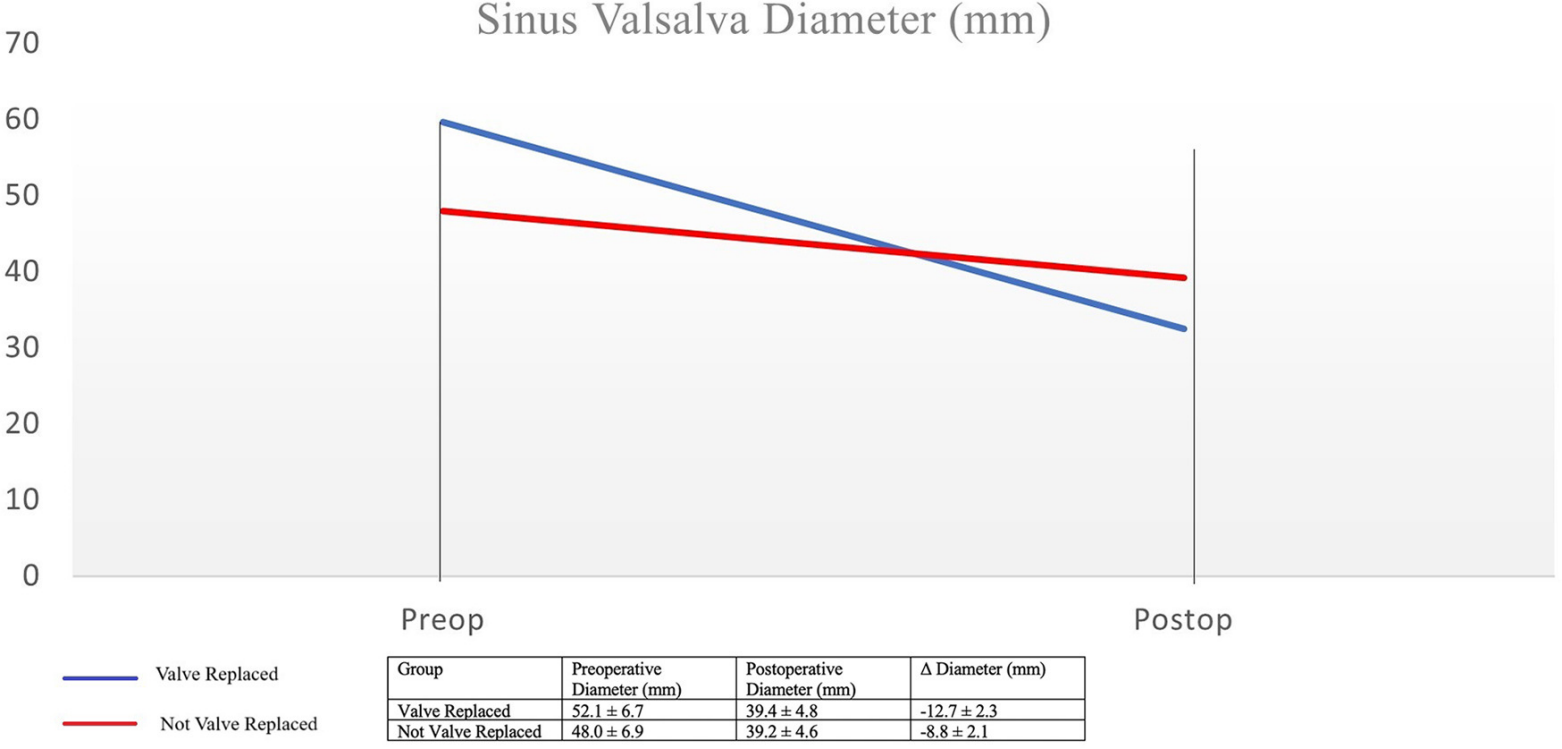
Sinus valsalva diameter.

Contrast CT angiograms were examined. The control time for computed tomography in postoperative follow-ups was found to be 21.0 ± 12.6 months. Measurements were taken from various parts of the patient's aorta on CT scans, including the sinus of Valsalva, pulmonary trunk, aortic arch, proximal thoracic aorta, mid-thoracic aorta, coeliac artery, superior mesenteric artery, renal arteries, infrarenal aorta, and iliac bifurcation. Postoperatively, due to the course of operations for both groups, sinus of Valsalva and pulmonary trunk diameters significantly decreased (*p* < 0.05) compared to preoperative values. Increase in diameter was seen in all other parts of the aorta, distal to the aortic arch, in the postoperative period compared to preoperative measurements (*p* < 0.05). In comparing the groups, the diameter of sinus of Valsalva in the postoperative period was observed to be lower in the the valve replaced group (*p* < 0.05). Despite this result, no sinus of Valsalva aneurysm was detected in any patient the not valve replaced group (Preoperative: 48.0 mm ± 6.9 mm, Postoperative: 39.2 mm ± 4.6 mm). Although statistically insignificant, when the increase in diameters in the distal aortic parts is examined, the increase in curve rates is in favor of the valve replaced group (Figure 1).

**Figure 2:**
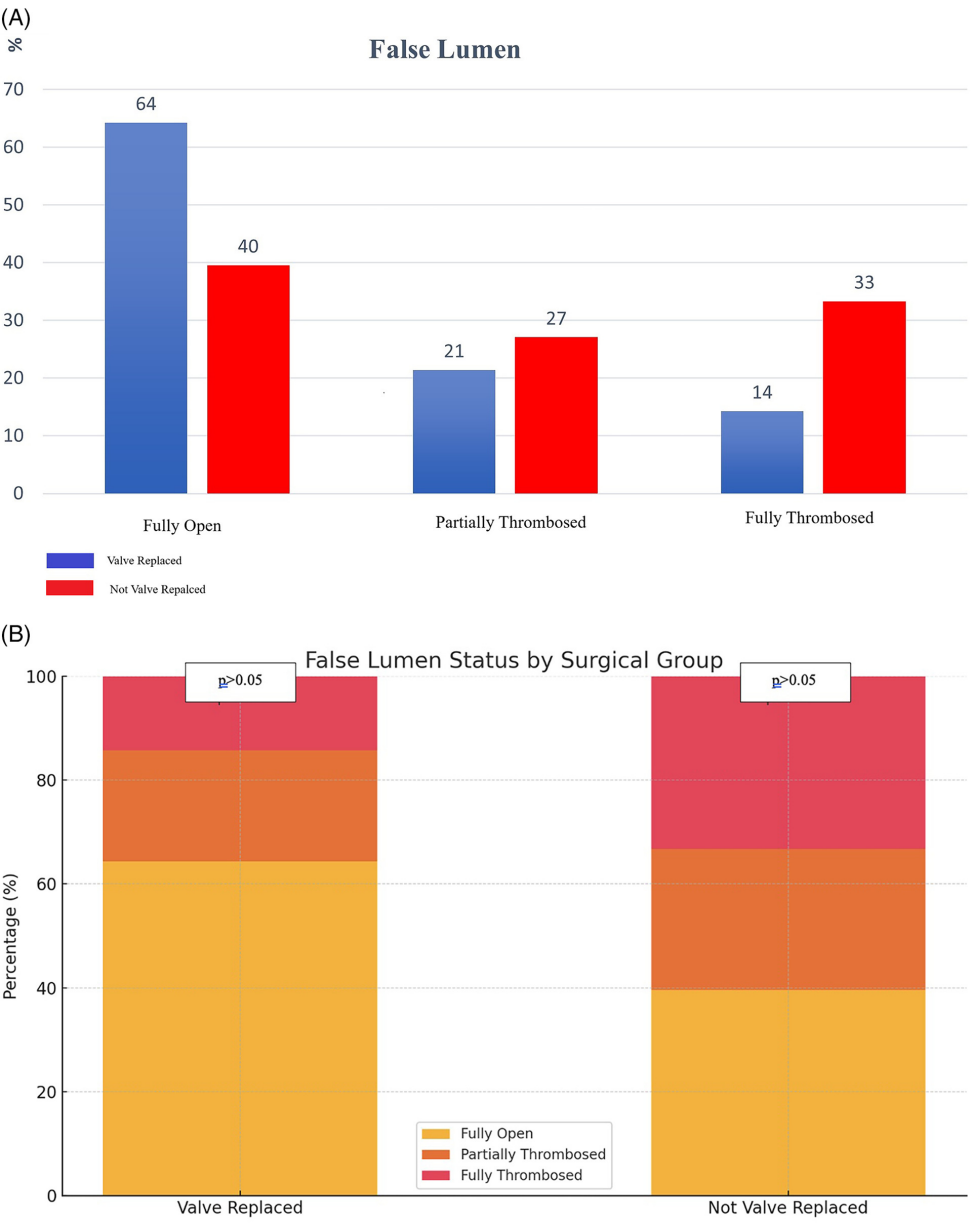
**(A)** False Lumen Status by Surgical Group. **(B)** False Lumen Status by Surgical Group.

The false lumen in the distal aorta was categorized into three groups: fully open, totally thrombosed, and partially thrombosed. The fully open false lumen rate was found to be 64.3% in the valve replaced group, while this rate was 39.6% in the not valve replaced group. The totally thrombosed false lumen rate was 14.3% in the valve replaced group and 33.3% in the not valve replaced. (*p* < 0.05) (Figure 2).

In nearly all patients, to thoroughly examine the length of the dissection flap and improve the quality of distal repair, the repair was achieved using the open anastomosis technique via antegrade selective cerebral perfusion, which is accepted as a safe method. Therefore, the right high brachial artery or the right axillary artery were mainly used as arterial cannulation sites. If these cannulation sites were not suitable, or if it was thought that full flow could not be provided for the operation, femoral arterial cannulation was preferred alone or in combination with axillar cannulation (*n* = 4, 3.6%). Only 4 patients (3.6%) needed repair postoperatively due to the cannulation site. No ischemic complications were observed in follow-ups of these patients. During Antegrade Selective Cerebral Perfusion (ASSP), patients were followed with Near-Infrared Spectroscopy (NIRS) to protect against the risk of neurological complications. The adequacy of the flow from the left CCA was checked during the operation. A total of three patients were thought to have inadequate flow leading to the left CCA being perfused by a second cannula during the repair, providing bilateral circulation.

In one patient with aberrant right subclavian artery and one patient with total occlusion of the left CCA, repair was performed under total circulatory arrest (18°C). Except for these 2 patients, all operations were performed under moderate hypothermia (26°C–28°C).

All patients were subject to topical cooling alongside antegrade, retrograde, and selective cardioplegia via coronary buttons as cardiac conservation methods. Del Nido was used for cardioplegia in 75.9% (*n* = 85) of patients. Other patients used HTK (Custodiol) (13.4% *n* = 15), Plejisol (9.8% *n* = 15), and Microplegia (0.9% *n* = 1).

In the valve replaced group, X-Clamp times and CPB times were found to be significantly longer (*p* < 0.001). X-Clamp times for the valve replaced group were 158.4 min ± 43.4 min compared to 107.1 min ± 35.8 min within the group that received the not valve replaced (Table 4).

**Table 4:** Intraoperative parameters.

	The Valve Replaced group	The not Valve Replaced group	*P* *
	(*n* = 26)	(*n* = 86)	
X-Clamp time (min) (25/86)	158.4 ± 43.4	107.1 ± 35.8	<0.001
CPB time (min) (24/82)	218.2 ± 50.2	166.2 ± 51.5	<0.001
ASSP (min) (22/74)	37.1 ± 28.7	38.0 ± 18.2	0.893
ASSP Bilateral (min) (0/3)	–	65.0 (44.0–77.0)	ns
TCA (min) (0/3)	–	40.0 (33.0–45.0)	ns

* Independent Samples *t* Test (Mean ± SD).

In total, 14% of patients (*n* = 16) underwent an additional surgical procedure. The majority (*n* = 13) of these additional surgical procedures were coronary artery bypass grafts. CABG requirements were determined intraoperatively when the dissection flap extended to the mouth of the right or left coronary button or when ventricular dysfunction was observed during weaning from cardiopulmonary bypass, in anticipation of possible coronary artery disease. Mitral Valve Replacement (MVR) was performed in 3 patients due to moderate-severe mitral regurgitation as per preoperative echocardiography. Secundum ASD repair was performed in one patient, and left femoral embolectomy for fenestration purposes was performed in one patient. No significant difference was found between the two groups in regard to necessity for an additional procedure.

Postoperative inotropic need was evaluated according to Vasoactive Inotropic Score (VIS) (10). Postoperative inotropic need was present in 73.2% (*n* = 82) of all patients, with no significant difference between the two groups (*p* > 0.05). Nonetheless, the requirement for mechanical support devices (ECMO/IABP) was significantly greater in the valve replaced group (*p* < 0.05). 3 patients (11.5%) in the valve replaced group required ECMO, and 1 patient (3.8%) required IABP due to cardiac failure. In the other group, only 2 patients (2.3%) were in need of ECMO.

The distal repair was performed under the closed anastomosis technique in 7 patients where the dissection was limited to the ascending aorta preoperatively (Stanford Type 2). 85 patients required HAR and 20 patients required TAR. There was no statistical difference in TAR or HAR between the two groups (*p* = 0,614). Patients who received Total Arch Replacement (TAR) and Hemi Arch Replacement (HAR) were compared. It was observed that the overall and early death rates were significantly higher in the TAR group, and time of death was significantly earlier (*p* < 0.05). For postoperative complications, peripheral ischemia and arrhythmia rates were found to be significantly higher in the TAR group. The rate of SVO was 25% in the TAR group and 12.9% in the HAR group (*p* = 0.182).

A total of 35 patients (31.3%) died in the postoperative period. Deaths occurring in hospital or within the first month were considered early deaths. The majority of these patients (*n* = 27, 24.1%) died early, and among the early deaths, the most common cause (*n* = 16) was related to postoperative cardiac conditions. The early death rate was 30.8% (*n* = 8) in the valve replaced group, and 22.1% (*n* = 19) in the not valve replaced group. There were 8 cases of late death. The late death rate was 3.8% (1 patient) in the valve replaced group, and 8.1% (7 patients) in the not valve replaced group. The time of death was determined to be 96.6 ± 231.5 days (Median 6.0 days (min 0.0—max1.080.0). However, no statistically significant difference was found between the groups in terms of early and late mortality rates.

**Figure 3:**
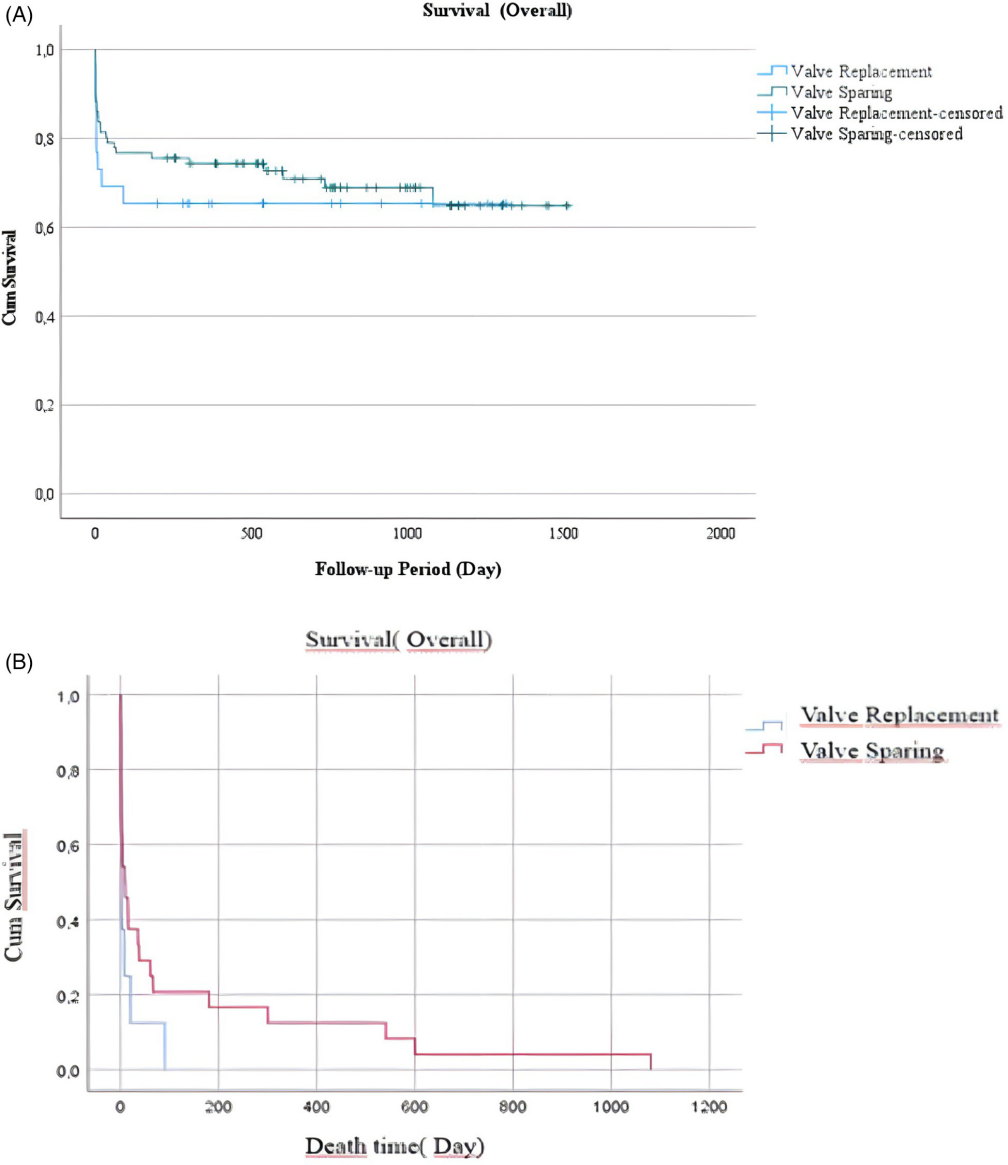
**(A)** Survival (Follow up period). **(B)** Survival (Death times).

When survival/death times were evaluated, both the overall, early, and late survival/death times of patients who the not valve replaced group were noticeably longer. The median follow-up time (21.0 ± 12.6 months) and median survival (overall: 1,067.8 days). The death times were found to be 15.9 days in the valve replaced group and 123.5 days in the not valve replaced group. The survival times were found to be 864.7 days in the valve replaced group and 1,067.8 days in the not valve replaced group However, no statistically significant difference was found due to the distribution of the patients (Figure 3).

No patient required reoperation for the proximal aorta or aortic valve. Five patients required reoperation for the distal aorta. Among these patients, an aorto-aortic tube graft operation was performed on one young Marfan patient with a descending aortic aneurysm. In one elderly patient, with a similar descending aortic aneurysm, aortic endovascular repair was performed after carotid-subclavian bypass. A third patient who returned with an aneurysm in the aortic arch and descending aorta after isolated ascending aortic replacement with the closed anastomosis technique during the first operation, required debranching + Thoracic Endovascular Aortic Repair (TEVAR). However, this patient died before TEVAR folloving debranching surgery. One other patient with a saccular aneurysm in the aortic arch and one patient with a Crawford Type V thoracoabdominal aortic aneurysm refused the operation and are therefore under observation. When comparing groups, no statistical difference was found in terms of the need for reoperation.

## Discussion

Despite a consensus on the emergency necessity of surgical intervention for acute Type A Aortic Dissection, there is an ongoing debate regarding the optimally inclusive approach for the surgical procedure. The procedure can manifest in a myriad of combinations across a broad spectrum (11). One critical consideration is whether or not to intervene with the aortic valve and the aortic root. This potential intervention arises mainly in patients whose aortic leaflets are neither entirely healthy nor irretrievably destroyed. In scenarios where aortic regurgitation is involved, studies cite the safe application of preserving the native aortic valve using AV resuspension or isolated supracoronary graft interposition in cases where the aortic root has not dilated, and the intimal flap has remained on the sinotubular junction (4–6, 12, 13). Our study found that even in patients with moderate to severe preoperative aortic regurgitation, none of those patients who underwent valve-sparing surgery experienced serious postoperative aortic regurgitation. This result supports the hypothesis that preserving the native valve is a safe path when the valve has not been completely damaged. This choice also substantially reduces operation and x-clamp times, thus minimizing potential intraoperative and postoperative adverse outcomes.

Another concern with proximal repair is the possibility of recurrent dissection or aneurysm development in the tissue of sinus valsalva, preserved in some cases. However, in our study, no sinus valsalva aneurysms were observed in any of the patients in the majority group who underwent an isolated supracoronary graft interposition, thus alleviating these concerns.

In the section of the aorta that did not undergo an operation, some studies have found that a higher percentage of residual false lumen opening can lead to poorer long-term survival and higher rates of reoperation (14–16). In our study, the rate of the entirely open false lumen was higher in the group that the valve replaced group, while the rate of the entirely thrombosed false lumen was higher in the not valve replaced group. The aortic diameter growth curve was higher in the group that underwent valve replacement based on measurements made at various points in the distal aorta. This situation might be associated with the mandatory use of anticoagulants in the postoperative period in patients who underwent valve replacement.

Acute TYPE A AORTIC DISSECTION, even when operated on by the most experienced surgeons at the most experienced centres, is associated with a high mortality rate (17). The mortality rate that emerged from this study is comparatively high in line with the literature when compared with other cardiac surgeries. The time of death was earlier in the group that the valve replaced group. Also, the need for mechanical support was higher in this group. These results can be interpreted as indicating that valve replacement in aortic dissection surgery yields less favourable results in terms of ensuring survival.

Findings from this study indicate that while the type of surgery (valve replacement or not) can influence some postoperative outcomes such as X-Clamp and CPB times, inotropic use, and false lumen rate, other factors such as echocardiographic findings, overall death rates, and ICU stay durations seem unaffected. In terms of the survival/death times, although the not valve replaced group had longer survival times in general, no statistically significant difference was found due to the distribution of the patients. These findings highlight the complex considerations involved in surgical decisions and underscore the necessity for personalized treatment approaches based on patients' unique circumstances and needs. Further research may allow for development of improved prediction models to optimize surgical decision-making and patient outcomes in cases of aortic dissection and replacement.

## Limitations

The retrospective design of the study is a limitation. Another limiting factor is the low number of cases in subgroups due to our center being relatively new. Hence, the follow-up period and the number of patients are limited to those operated on since the hospital's establishment date in February 2019. These aspects mark the significant constraints of this study.

## Conclusion

Acute TYPE A AORTIC DISSECTION constitutes one of the cardiovascular pathologies that present with high perioperative risk. Successful surgical intervention, in this case, is largely contingent on the rapid and accurate decision-making for patients in their preoperative and intraoperative stages. The findings in our study suggest that in the surgery of aortic dissection, the not valve replaced surgical methods can be securely performed. We demonstrated that even in patients presenting with moderate to severe preoperative aortic regurgitation, none of those subjected to the not valve replaced surgery experienced severe postoperative aortic regurgitation. This lends credence to the safety and efficacy of the not valve replaced techniques when the aortic valve is not entirely compromised.

Moreover, our study offers some reassurance regarding a common concern in proximal repair involving the possibility of recurrent dissection or aneurysm development in the tissue of the sinus valsalva. Specifically, sinus valsalva aneurysms were not observed in any patients who underwent isolated supracoronary graft interposition.

In light of these findings, although the not valve replaced methods emerge as a safe alternative approach in aortic dissection surgery, it becomes aligned with the need for further research. Specifically, a more robust, multicenter evaluation involving larger patient populations and longer follow-up periods is essential to corroborate these findings conclusively and inform future practice.

## Data Availability

The original contributions presented in the study are included in the article/Supplementary Material, further inquiries can be directed to the corresponding authors.
